# Case report: Clinical management of recurrent small cell lung cancer transformation complicated with lung cancer-induced acute pancreatitis after lung adenocarcinoma surgery

**DOI:** 10.3389/fphar.2023.1259221

**Published:** 2023-10-31

**Authors:** Suyun Zhang, Ningjing Guo, Qianyuan Zhang, Yao Wang, Sheng Yang, Xiangqi Chen

**Affiliations:** ^1^ Departments of Internal Medicine, Fujian Medical University Union Hospital, Fuzhou, Fujian, China; ^2^ Oncology Medicine, Fujian Medical University Union Hospital, Fuzhou, Fujian, China; ^3^ General Medicine, Fujian Medical University Union Hospital, Fuzhou, Fujian, China; ^4^ Respiratory Medicine, Fujian Medical University Union Hospital, Fuzhou, Fujian, China

**Keywords:** lung adenocarcinoma, small cell lung cancer, acute pancreatitis, pathological type transformation, case report

## Abstract

In the diagnosis and treatment of non-small cell lung cancer (NSCLC), the histological type may change from lung adenocarcinoma to lung squamous cell cancer or small cell lung cancer (SCLC). Pancreatic metastasis is extremely rare in advanced lung cancer, and pancreatitis characterized by lung cancer metastasis-induced acute pancreatitis (MIAP) is more rare. This paper reports in detail the clinical diagnosis and treatment of a female patient with lung adenocarcinoma who relapsed after radical surgery and progressed after multiple treatments. A second pathological biopsy revealed SCLC transformation, and the patient developed pancreatic metastasis and lung cancer MIAP during follow-up treatment. This paper mainly suggests that clinicians should pay attention to the possibility of pathological type transformation in the progression of advanced NSCLC, closely observe the dynamic changes of tumor markers and pay attention to the re-biopsy pathological analysis. In addition, it provides clinical experience and scientific reference for the discovery, diagnosis and treatment of transforming SCLC and lung cancer MIAP.

## Brief medical history

**FIGURE 1:**
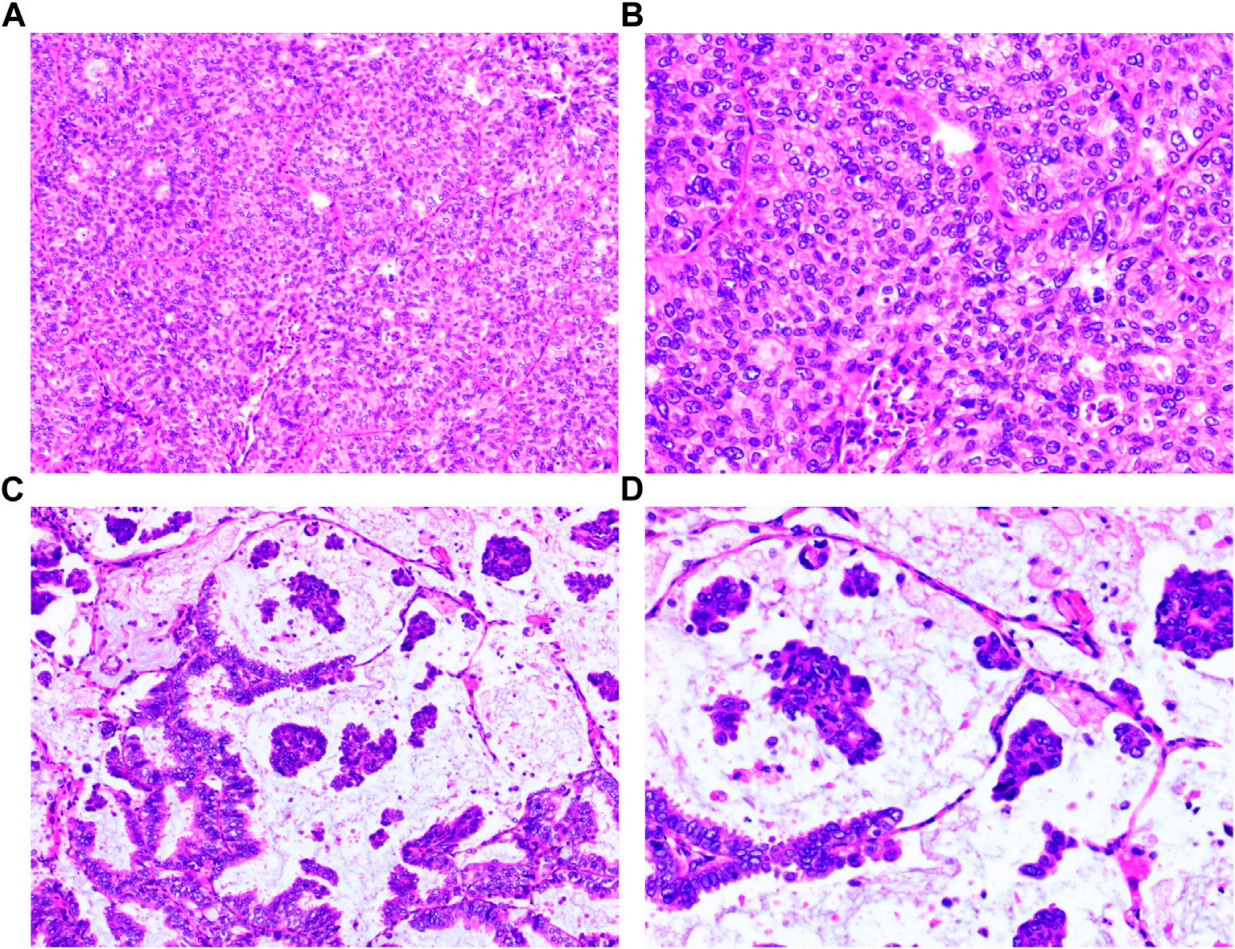
Histopathology of the surgical tumor. **(A)** Invasive adenocarcinoma (HE: ×100); **(B)** Invasive adenocarcinoma (HE: ×200); **(C)** Mucinous adenocarcinoma (HE: ×100); **(D)** Mucinous adenocarcinoma (HE: ×200).

A 58-year-old female patient was referred to the Department of Thoracic Surgery of Fujian Medical University Union Hospital due to “a lung nodule found in the left lower lobe” during a physical examination at the local Hospital A in Fujian Province, China on 23 March 2016. After various examinations, a main clinical diagnosis was made that confirmed the presence of a lung nodule in the left lower lobe. However, to determine whether this nodule is a malignant lung tumor, further examinations were required. After active preparation, the patient underwent “thoracoscopic left lower lobectomy + systematic mediastinal lymph node (LN) dissection” under general anesthesia on 30 March 2016. The postoperative pathological diagnosis was as follows: The size of the lesion in the left lower lobe was 2.5 cm × 2.3 cm × 2.0 cm, including 70% of invasive adenocarcinoma and approximatively 30% of mucinous adenocarcinoma (Figure 1) that did not involve the visceral pleura. No metastases were detected in all groups of LNs. Immunohistochemical results indicated adenocarcinoma that was diffuse positive for NapsinA, TTF-1, CK7, and SP-B, partially positive for CK20, negative for CDX2, and that has an epidermal growth factor receptor (EGFR) exon 19del. The postoperative pathological stage was pT1cN0M0, stage Ia3. After discharge from the hospital, the patient was followed up through regular outpatient reviews. On 6 April 2017, the patient was reexamined at the Outpatient Department of Fujian Medical University Union Hospital. There was no abnormality after physical and imaging examinations, but the level of the blood tumor marker, carcinoma embryonic antigen (CEA) (6.9 ng/L), was slightly higher than the normal value (<5 ng/L). CEA level gradually rose during the close follow-up, and the patient chose to take first-generation TKI gefitinib for prevention and treatment, but CEA still gradually increased, so she switched to third-generation TKI osimertinib. Whole-body positron emission tomography-computed tomography (PET-CT) examination was performed at the Outpatient Department of Fujian Medical University Union Hospital on 13 October 2017, indicating the presence of multiple LN metastases in the mediastinum, bilateral supraclavicular, and infraclavicular regions, and bilateral neck. At the same time, the CEA level was as high as “14.6 ng/L.” The patient was admitted to the Department of Thoracic Surgery on 26 October 2017, and then received “EBUS-TBNA” under general anesthesia on 2 November 2017, however, no cancer cells were discovered in the postoperative pathology. Therefore, the patient was referred to the First Affiliated Hospital, Zhejiang University School of Medicine where she underwent “puncture of the right supraclavicular mass” at the Outpatient Department on 26 November 2017. The immunohistochemical results of the punctured tissues showed adenocarcinoma with possible pulmonary origin.

## Treatment history

### Treatments before SCLC transformation

The patient was readmitted at the Department of Thoracic Surgery of Fujian Medical University Union Hospital on 15 December 2017 due to the presence of multiple LN metastases in the mediastinum, bilateral supraclavicular and infraclavicular regions and bilateral neck after radical surgery for invasive adenocarcinoma in the left lower lobe. As the stage of the punctured tumor tissues was not sufficient to support the detection of related lung cancer (LC) driver genes, a hematological test was performed, and the results were negative. The patient was then treated with a regimen including “pemetrexed, cisplatin and bevacizumab at q21d” for 6 cycles from 2 January 2018 to 7 May 2018, and the comprehensive evaluation of efficacy was “stable disease (SD)” at the periodic reexamination. However, the whole-body CT examination at the Outpatient Department of the hospital on 28 June 2020, indicated that the mediastinal LNs and the right axillary LNs are significantly larger than before, and the comprehensive assessment of the efficacy was “progressive disease (PD).” Through multidisciplinary discussion, the following main opinion was made: The patient’s cancer is significantly developed, and there are indications for continuing palliative chemotherapy and local radiotherapy. The patient accepted a 10-cycle treatment regimen of “albumin paclitaxel at q21d,” combined with “antiangiogenic bevacizumab/PD-1 inhibitor tislelizumab” at the Department of Thoracic Surgery of Fujian Medical University Union Hospital from 13 June 2020 to 23 March 2021. Moreover, the patient received “Cyberknife” radiotherapy at the Radiotherapy Department from 2 February to 17 February 2021.

### The detection of SCLC transformation

**FIGURE 2:**
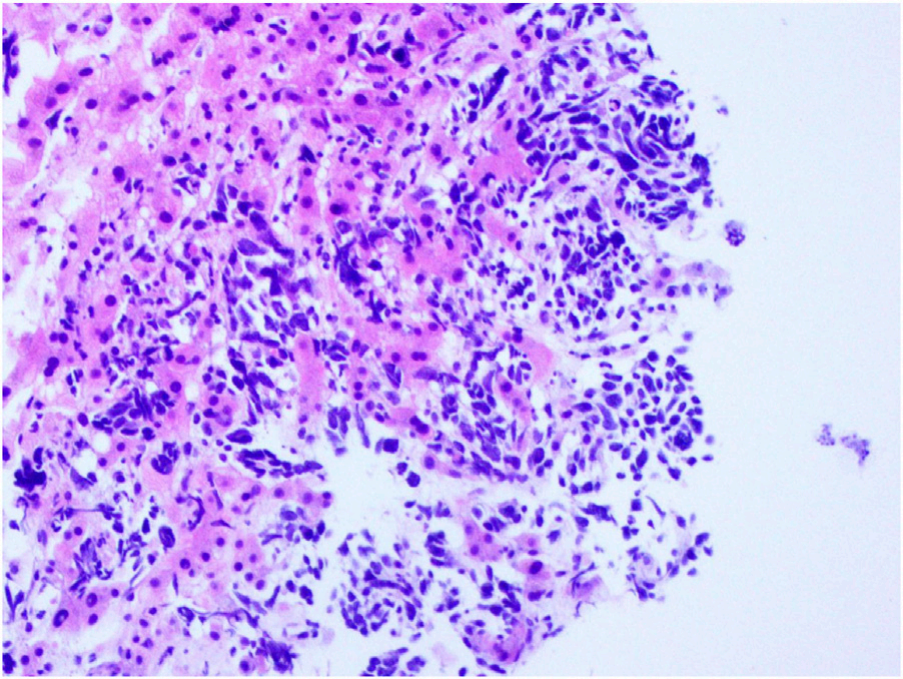
Biopsy pathology of liver mass: small cell cancer (HE: ×200).

On 17 March 2021, the patient underwent a second whole-body CT examination at the Outpatient Department of Fujian Medical University Union Hospital, which indicated the presence of multiple small enlarged LNs in the left neck, mediastinum, right supraclavicular region, and axillary fossa shrank. There were multiple nodes with abnormal signals in the liver and behind the peritoneum, which were metastatic. The comprehensive assessment of efficacy was “progressive disease (PD)” due to “new liver lesions.” Additionally, several hematological tumor markers were found changed during this reexamination. Specifically, progastrin-releasing peptide (proGRP) raised to “287 pg/mL (normal value < 69.2 ng/L)” and neuron-specific enolase (NSE) increased to “27.29 ng/mL” while CEA decreased to “9.5 ng/mL.” The primary opinion made *via* a multidisciplinary discussion among practitioners at the outpatient departments of thoracic surgery, respiratory medicine, radiotherapy, and medical oncology was as follows: There is a high probability of transformation of lung adenocarcinoma into small cell lung cancer (SCLC), and therefore, a puncture biopsy of the liver metastases is recommended. The patient was admitted to the Department of Medical Oncology of Fujian Medical University Union Hospital and underwent CT-guided puncture of liver metastasis on 25 March 2021. The immunohistochemical results showed small cell cancer (Figure 2). The main clinical diagnosis was multiple metastases in the mediastinum, bilateral supraclavicular and infraclavicular regions, and bilateral cervical LNs, and liver metastases and SCLC transformation after radical treatment of invasive adenocarcinoma in the left lower lobe. From 03 November 2017 to 03 April 2021, this patient, with lung adenocarcinoma who relapsed after radical surgery, survived for 41 months after multiple treatments.

### Clinical management after SCLC transformation

She then received 2 cycles of chemotherapy with “etoposide + carboplatin at q21d” for advanced SCLC from 3 April to 25 April 2021. The comprehensive assessment of the efficacy was “SD” on 30 April 2021. On 8 May 2021, her brain magnetic resonance (MR) examination results suggested the existence of multiple intracranial metastases. After multidisciplinary discussions, she was recommended to accept radiotherapy for brain metastases and to change the systemic chemotherapy regimen. But, considering that intracranial metastases were relatively small and did not cause any brain discomfort, she requested to suspend radiotherapy, and to continue the “etoposide + carboplatin at q21d” chemotherapy, as there was a good control of the extracranial lesions by this regimen. After the 4th and 6th cycles, the comprehensive assessment of the efficacy was “SD”. However, later, on 20 October 2021, it turned into “PD”, as both intrahepatic and intracranial metastases were found to be larger and more numerous than before and higher levels of hematological tumor markers were tested (proGRP 798.20 pg/mL, CEA 126.7 ng/mL, and NSE 38.37 ng/mL). Therefore she started second-line therapy. From 26 October 2021 to 11 January 2022, four cycles of chemotherapy with “irinotecan at q21d” were selected, and whole-brain radiotherapy was performed during the chemotherapy interval. Radiotherapy was performed using IMRT with the whole brain as the clinical target volume (CTV), an external expansion of 0.3 cm as the planning target volume (PTV)-CTV, and a DT 30Gy/10F. On 16 December 2021, the comprehensive assessment of the efficacy was “SD”. However, it became “PD” on 10 February 2022 due to new metastases found in the liver, the right acetabulum, the right sacrum, and the left ilium. She was transferred to the third-line therapy, during which oral targeted therapy with “10 mg of Anlotinib Hydrochloride Capsules at d_1-14_ and q21d” was selected, and diphosphonate was used to resist bone metastasis as specified. Afterward, the disease kept relatively stable.

### The occurence of LC-induced MIAP

On 24 May 2022, she was admitted to our hospital due to a feeling of intermittent abdominal oppressive pain and discomfort, with the main clinical diagnosis of abdominal pain of undeterminded reason, SCLC with mutiple metastases (to the brain, liver, bones, and right supraclavicular and right axillary LNs), and recurrence of left lung adenocarcinoma after surgery. Following positive symptomatic treatment, the symptoms were slightly improved, and no obvious specific abnormalities were detected in the whole blood routine and biochemical tests. However, the comprehensive assessment of the efficacy was “PD” because of the increased size and number of intrahepatic metastases and higher levels of hematological tumor markers (proGRP 10550 pg/mL, CEA 146.8 ng/mL, and NSE 30.22 ng/mL). Besides, a acute pancreatitis (AP) with undefined cause was also discovered through plain + enhanced MR of the abdomen. On 29 May 2022, the patient’s blood amylase was 300 IU/L, the blood lipase was 5,396 U/L, and the urinary amylase was 2,240 IU/L. She was transferred to the Department of Gastroenterology for further diagnosis and treatment.

### Treatments mainly for LC-induced MIAP

**FIGURE 3:**
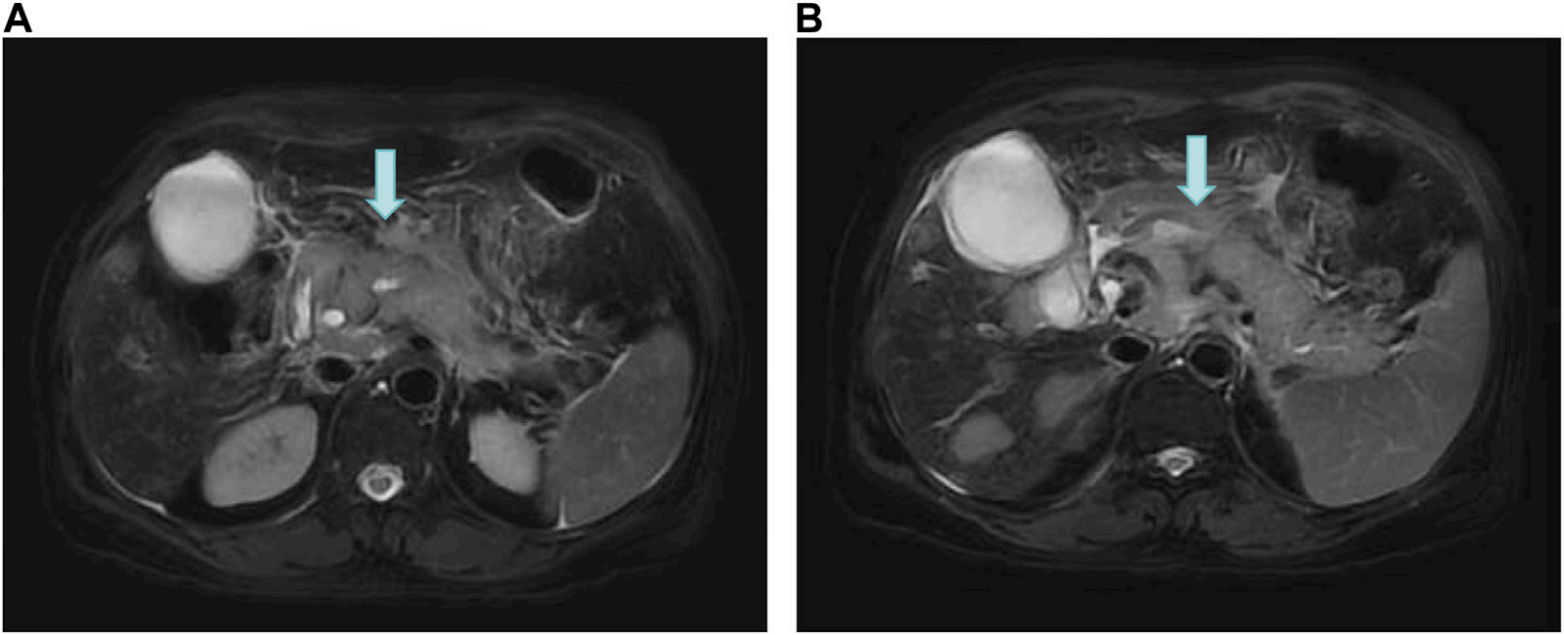
MR Examination of the pancreas. **(A)** Pancreatitis with peripheral exudation; **(B)** Multiple malignant tumors (possible metastases) of the pancreas.

**FIGURE 4:**
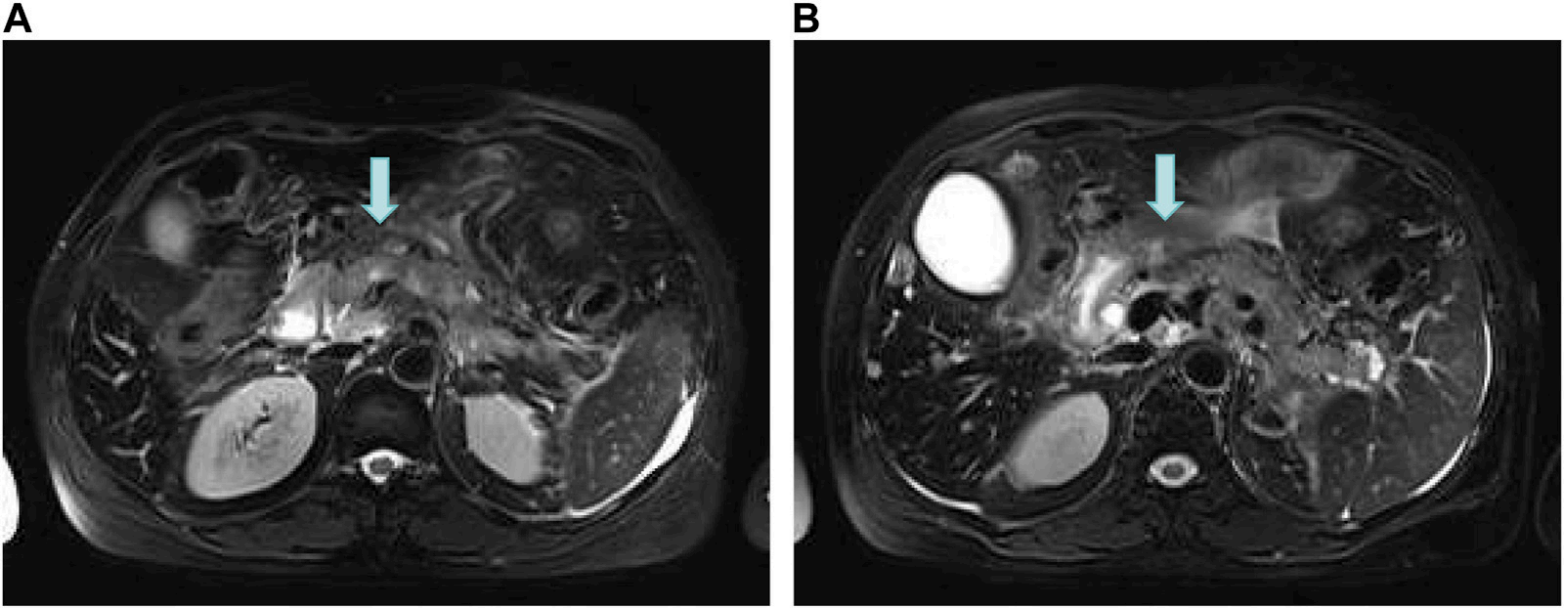
MR Examination of the pancreas. **(A)** Pancreatitis accompanied by peripheral exudation; **(B)** Multiple malignant tumors (metastatic tumor possible) of pancreas, smaller than before.

On 16 June 2022, the blood amylase was 592 IU/L, the blood lipase was 10,809 U/L, the urinary amylase was 10,112 IU/L, and the total bilirubin was 32.5 μmol/L (normal range: 2.0–22.0 μmol/L). Images from plain + enhanced CT of the whole abdomen (Supplementary Figure S1), magnetic resonance cholangiopancreatography (MRCP) and MR examination of pancreas (Figure 3) indicated acute pancreatitis accompanied by possible multiple pancreatic metastases. On 20 June 2022, She was diagnosed with postoperative recurrence of the left lung adenocarcinoma (mutiple metastases in the brain, liver, pancreas, bones, right supraclavicular region LNs, and right axillary fossa LNs), which was subsequently transformed into SCLC complicated with post-treatment LC-induced AP. Based on these observations, it was recommended that a comprehensive antitumor therapy should be carefully selected. On 23 June 2022, her growing blood amylase (874 IU/L), urinary amylase (24,705 IU/L), total bilirubin (150.2 μmol/L), and conjugated bilirubin (108.4 μmol/L, normal range: 0–8.0 μmol/L) indicated the complications of LC-induced MIAP and obstructive jaundice. Considering that inconspicuous intrahepatic bile duct dilatation was not suitable for PTCD surgery at that time, she was recommended to start with palliative radiotherapy for the pancreatic metastasis, followed by an antitumor drug therapy. The patient received five sessions of radiotherapy from 24 June 2022 to 1 July 2022, during which she was regularly treated with oral “imidazolotetrazine alkylating agents, temozolomide and pamiparib.” On 26 June 2022, the total blood bilirubin was 129.9 μmol/L, and the conjugated bilirubin was 95.5 μmol/L. On 29 June 2022, the blood amylase was 157 IU/L, and the urinary amylase was 1,322 IU/L. The patient’s condition was gradually improving. On 3 July 2022, the blood amylase was 85 IU/L, urinary amylase was 331 IU/L, the blood total bilirubin was 36.5 μmol/L, and the conjugated bilirubin was 24.0 μmol/L. On 4 July 2022, MR Examination of pancreas indicated that pancreatitis accompanied by peripheral exudation, multiple malignant tumors (metastatic tumor possible) of pancreas, smaller than before (Figure 4). On 5 July 2022, she was discharged due to disease improvement. Thereafter, she went to the local hospital for regular hematological examinations, which revealed gradual recovery to normal conditions without significant abnormalities.

On 9 October 2022, the patient was readmitted to our hospital for reexamination and the comprehensive assessment of the efficacy was “PD” due to the enlargement of metastases in liver and LNs and higher hematological tumor markers (proGRP 2165 pg/mL, CEA 926.5 ng/mL, and NSE 118.9 ng/mL). However, there were no specific abnormalities in the blood routine, the liver and kidney functions, and the levels of blood amylase, blood lipase, and urinary amylase. She started to receive the first cycle of chemotherapy with “paclitaxel at q21d” of the fifth-line therapy for SCLC and the first split-course radiotherapy, during which “the right neck + right supraclavicular LNs were selected as the GTV, with an external expansion of 0.5 cm as the PTV-GTV (treated with 5Gy/1F). The whole liver was also set as the GTV, with an external expansion of 0.5 cm as the PTV-CTV (treated with 5Gy/1F).” Besides, the targeted therapy combined with “poly-ADP-ribose-polymerase (PARP) inhibitor, pamiparib” was continued, the patient was discharged due to improvement on 3 November 2022. From 03 April 2021 to 30 November 2022, this patient had been treated for the transformed SCLC for 20 months. Thereafter, she went to the local hospital for further treatments and lost follow-up.

## Case discussion

Based on incidence and mortality rates, LC currently ranks top among the malignant tumors worldwide. This cancer seriously threatens human life and health (Bray et al., 2021). LC can be divided into non-SCLC (NSCLC) and SCLC according to pathological types, of which the former accounts for approximately 85% of diagnosed LCs, which mainly includes adenocarcinoma, squamous cell carcinoma, and large cell cancer. For inoperable driver gene-positive NSCLC, targeted therapy is the preferred strategy, while simple chemotherapy is the cornerstone treatment for NSCLC without driver alterations. Simple chemotherapy can be combined with anti-angiogenic/immunotherapy or radiotherapy (Ettinger et al., 2019). Numerous studies have shown that resistance to first-line antineoplastic drugs for advanced NSCLC mostly occurs at 6–12 months. The resistance to the first use of antineoplastic drugs is known as primary or intrinsic resistance, while resistance that emerges during treatment is known as acquired or secondary resistance (Han et al., 2017). It has been found that the mechanism of chemoresistance is closely related to abnormal activities of multidrug resistance genes and mitochondrial signaling pathways, which involve the effects of multiple factors including chromosome abnormality in tumor cells, reduced intracellular drug accumulation, enhanced DNA damage repair function, strengthened cellular detoxification function, apoptosis inhibition, abnormal vascularization, cytoskeleton abnormality, and abnormal density of extracellular peripheral matrix. However, the specific mechanism of chemoresistance is still unclear and more detailed mechanisms need to be explored. Ferrer et al. (2019) retrospectively analyzed post-treatment clinical data on the transformation of wild-type NSCLC into SCLC in 10 adenocarcinoma patients and 3 squamous carcinoma patients (EGFR) and found that among the SCLCs patients who were treated with second-line therapies on average at the time of pathological transformation, more than 85% received etoposide combined with platinum-based chemotherapy regimens and that the overall survival was approximately 37 months. Previous studies suggested that the treatment pressure of chemotherapy or radiotherapy rarely leads to the transformation of NSCLC to SCLC (Marcoux et al., 2019). In 2006, Zakowski et al. (2006), at the Memorial Sloan-Kettering Cancer Center, first reported a case of a 45-year-old female patient with lung adenocarcinoma who suffered disease progression after EGFR-tyrosine kinase inhibitor (TKI) treatment and chemotherapy, and which biopsy pathology showed synaptophysin-positive SCLC. Since then, SCLC transformation has been gradually recognized, and studies have uncovered that 5%–15% of EGFR-mutated NSCLCs undergo SCLC transformation during the disease evolution. However, studies have also found that SCLC transformation commonly occurs in patients with EGFR-mutated NSCLC progression after TKI treatment, and that SCLC transformation may also occur following antineoplastic drug treatment for fusion mutation of anaplastic lymphoma kinase (ALK), c-ROS proto-oncogene 1 receptor tyrosine kinase (ROS1)-fused adenocarcinoma, EGFR wild-type lung adenocarcinoma, and lung squamous cell carcinoma (Caumont et al., 2016; Sehgal et al., 2020).

In this case, the patient was diagnosed with systemic multiple metastases after lung adenocarcinoma surgery through imaging and pathological puncture biopsy, and received “pemetrexed” and “albumin paclitaxel” chemotherapy as the main second-line therapy. After combination with radiotherapy, the biopsy indicated the occurrence of non-driver gene-positive advanced adenocarcinoma with SCLC transformation, and this was in the absence of targeted therapy using TKI drugs. In the retrospective analysis of Ferrer et al. (2019), SCLC transformation occurred in 13 cases of post-treatment EGFR wild-type NSCLC, which is a more realistic reflection of the clinical diagnosis and treatment, and different molecular features and treatment processes that may lead to a rare transformation, including SCLC. Many studies have argued that the transformation of pathological types may be a true phenotypic transformation, but it may also result from a malignant biological activity of mixed LCs containing both SCLC and adenocarcinoma, during which, the latter transforms into LC, dominated by post-treatment SCLC (Ahmed et al., 2018). Thus, the changing NSCLC types should be alerted during treatment despite the original pathological type, and the objective existence of complexity of LC pathology. Spatial and temporal heterogeneities and clonal subtypes should also be fully considered (Pignataro et al., 2020). Therefore, during clinical management, the dynamic changes of pathological types at the primary lesion, especially at the metastasis, should be considered in the case of rapid progression of advanced NSCLC or failure of multiline therapy during antitumor drug therapy. Additionally, the time of transformation of blood tumor markers, should also be considered. In this case, the early diagnosis of SCLC transformation was determined relatively early mainly due to the continuous monitoring of NSCLC and SCLC markers’ transformations. For example, it was discovered that proGRP is abnormally elevated, which is worth considering for when performing another biopsy in clinical management. To date, most studies have concluded that the treatment of transformed SCLC is similar to that of classic SCLC. In fact, additional in-depth studies and new strategies, should be explored for the diagnosis and treatment of LC transformed pathological types, and stronger clinical trial evidence should be obtained to support relevant clinical decisions (Roca et al., 2017). Meanwhile, it is noteworthy to highlight that TP53 gene mutation, loss of RB gene function, FHIT methylation and other phenotypes, are mostly detectable in SCLC tumor tissue specimens. However, such genetic phenotypes are poor prognostic factors for advanced NSCLC, just like a TP53 non-breaking mutation, and there are no effective targeted therapies (George et al., 2015; Wu et al., 2016; Hayashi et al., 2020). Therefore, more attention should be paid to the potential SCLC transformation in such NSCLC patients. It is also suggested that a timely monitoring of gene mutations for timely adjustment of treatment regimens, should also be an important investigative direction for patients with post-treatment sudden progression.

In this case, the patient was diagnosed with systemic multiple metastases after lung adenocarcinoma surgery through imaging combined with pathological puncture biopsy (October 2017). The patient received “pemetrexed” and “albumin paclitaxel” chemotherapy as the main second-line therapy. After combined radiotherapy, another biopsy was conducted. Approximatively 41 months later, SCLC transformation occurred (March 2021). Thereafter, the patient was diagnosed and treated by reference to the specifications, guidelines, and consensus for the diagnosis and treatment of extensive-disease SCLC for approximatively 21 months. During the process, the patient developed AP in April 2022, which was timely detected with the support of a multidisciplinary team and controlled by scientific diagnosis and treatment strategies. This approach laid a good foundation for further improving the patient’s quality of life and prolonging survival. AP is a common disease in gastroenterology, with an incidence of approximatively 13–45/100,000, and that shows a continuously increasing trend. Approximatively 75% of AP cases are caused by cholelithiasis, hyperlipidemia, and alcohol abuse, while other rare causes include surgery, trauma, and drugs with approximatively 10% of idiopathic AP cases (Wu et al., 2017). Metastasis-induced AP (MIAP) is relatively rare and may be the primary or secondary clinical manifestation of a tumor, and therefore, it is often undetected leading to a poor prognosis. AP can be classified as mild AP (MAP), moderately severe AP (MSAP), and severe AP (SAP) according to the 2012 Atlanta criteria (Banks et al., 2013). The incidence rate of pancreatic metastasis in LC patients is low with approximatively 0.12%–7.50%. However, SCLC metastasis to the pancreas in SCLC autopsy reports, is not negligible as it accounts for approximatively 24%–40% (Wood et al., 2018). Studies have found that cases of LC-induced MIAP mostly have atypical symptoms, with mild or no symptoms. A small number of patients with pancreatic metastases manifest abdominal pain, AP, and obstructive jaundice. These patients often miss the timing of anti-lung cancer treatment due to the relative lack of a certain degree of clinical vigilance. The early detection of LC-induced MIAP is more conducive to improving LC prognosis. It has also been shown that patients with LC-induced MIAP are older and more vulnerable to anemia, main pancreatic duct dilatation, and abdominal LN enlargement than those with non-tumor-related AP (Gonlugur et al., 2014; Yamanashi et al., 2015). Xiong et al. (2020) reported that 7 out of 8 patients (87.5%) with LC-induced MIAP have a SCLC pathological type. Tanaka et al. (2009) retrospectively summarized 25 cases of LC-induced MIAP, including 19 (76.0%) cases of SCLC. Maeno et al. (1998) reported that the common pathological type of LC-induced pancreatic metastasis is SCLC, accounting for approximatively 50%, followed by adenocarcinoma (34.6%), squamous cell carcinoma (11.5%), and large cell lung cancer (3.9%). Besides, the proportion of MIAP in SCLC-induced pancreatic metastasis is high, which should be emphasized by clinicians. To date, there are few reports of LC-induced MIAP in both individual and multiple cases. In this case, the patient was diagnosed with the NSCLC transformation into SCLC, namely, SCLC accompanied with MIAP. The diagnosis was mainly confirmed by abdominal CT, MRI and ERCP examinations due to the patient’s abdominal pain, and the abnormal increases in blood amylase, blood lipase, and urinary amylase. Moreover, jaundice was gradually worsening, with a maximum level of total bilirubin of 149.2 μmol/L (normal range: 2.0–22.0 μmol/L). The patient’s clinical experience of MIAP is consistent with that of the results of related previous studies. It is believed that LC-induced MIAP may have multiple pathogeneses: 1) Mechanical compression of the pancreatic ducts by metastatic tumors or enlarged peripancreatic LNs; 2) Ischemia-induced pancreatic injury due to tumor invasion of blood vessels; and 3) Diffuse tumor infiltration to the pancreas and destruction of the pancreatic lobules. Studies have also observed that patients with LC-induced MIAP are mostly at an advanced stage at the time of detection, generally associated with poor conditions and high risks of surgical resection or puncture biopsy of pancreatic lesions or abdominal occupancies. Besides, such patients are often less willing to have a biopsy, and thus, the metastatic lesions of most these patients are clinically diagnosed by imaging findings (excluding AP induced by other reasons) (Okutur et al., 2015). It was reported that the treatment strategy for LC-induced MIAP mainly favors symptomatic and supporting treatment at the initial stage, and that MAP patients should start chemotherapy and/or radiotherapy as early as possible, to facilitate AP recovery. SAP patients may not be able to tolerate chemotherapy due to their poor physical conditions (Okutur et al., 2015). In this case, the LC patient developed MAP during treatment, which was improved with pharmacological chemotherapy combined with local radiotherapy and without AP recurrence. The patient has a good survival status with an ECOG score of 2 points at the follow-up to date, resulting in a prolonged median survival, compared to the extensive-disease SCLC reported in the National Comprehensive Cancer Network (NCCN) clinical practice guidelines. Of course, after postoperative, the recurrence of the left lung adenocarcinoma that subsequently transformed into SCLC complicated with LC-induced AP, was controlled. Statistics showed that a total of 5 lines of therapy were conducted after SCLC transformation, including chemotherapeutic agents “etoposide, irinotecan, cisplatin, carboplatin, temozolomide, and paclitaxel,” targeted agents “anlotinib (a new small molecule multi-target tyrosine kinase inhibitor), surufatinib (a selective tyrosine kinase inhibitor targeting vascular endothelial receptor (VEGFR) and fibroblast growth factor receptor (FGFR)), and pamiparib (a DNA repair enzyme (PARP) inhibitor).” Furthermore, a split-course radiotherapy, which significantly improved the patients’ quality of life and prolonged her survival, was also performed (Nyquist et al., 2020). In conclusion, the clinical symptoms of LC-induced MIAP are not specific, and the clinical types are mainly MAP, with poor prognosis, but an early diagnosis and a timely treatment may greatly improve the prognosis. For elderly patients, emaciation, or manifestation of other affected systems, anemia, main pancreatic duct dilatation, pancreatic occupancy and abdominal LN enlargement, are important clues for the diagnosis of LC-induced MIAP that require in clinical treatment.

The currently available clinical evidence indicates that SCLC has a shorter survival and fewer therapeutic options than NSCLC. As clinical studies have progressed, the transformation of SCLC pathological types is not uncommon under the pressure of therapeutic selection. Such pressure is not only attributed to the application of EGFR-TKI, but also involves that induced by chemotherapy. Moreover, whether the combination of anti-angiogenic, radiotherapy, immunotherapy, and surgery leads to the transformation of SCLC pathological types, needs to be confirmed by more relevant clinical studies. With the improvement of clinical practice skills and the rapid development of tumor molecular biology, the awareness of re-biopsy and dynamic genetic testing should be enhanced in the whole process of NSCLC multidisciplinary management of NSCLC to formulate the best treatment strategy for patients with greater precision (Zhu et al., 2019). Of course, for complications that are still relatively uncommon and rare, it is necessary to pay a high attention to the thought, and a timely and early detection, and a correct and active management. Additionally, attention should be paid to prevention in the clinical practice of overall tumor treatment. In conclusion, there is still a long way to go in investigating the molecular mechanisms of the occurrence and development of rare and uncommon complications during the diagnosis and treatment of the transformation of NSCLC into SCLC that requires more effective treatment strategies.

## Data Availability

The raw data supporting the conclusion of this article will be made available by the authors, without undue reservation.
