# Supplementary figures and images for: Case report: Clinical management of recurrent small cell lung cancer transformation complicated with lung cancer-induced acute pancreatitis after lung adenocarcinoma surgery
Source: Front Pharmacol. 2023 Oct 31;14:1259221. doi: 10.3389/fphar.2023.1259221 (PMC10644230; doi:10.3389/fphar.2023.1259221)

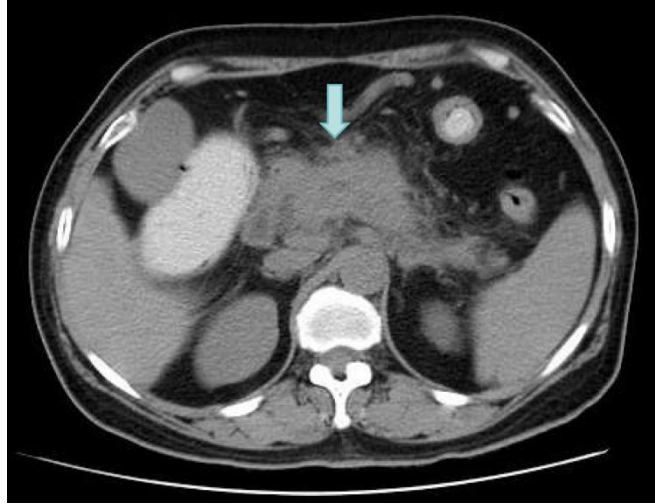

**Supplementary Figure 1. Plain + enhanced CT of the whole abdomen**

Supplement: Supplementary file 1 [file DataSheet1.PDF]
